# Optimizing ChIRP-MS for Comprehensive Profiling of RNA-Protein Interactions in *Arabidopsis thaliana*: A Telomerase RNA Case Study

**DOI:** 10.3390/plants13060850

**Published:** 2024-03-15

**Authors:** Lucie Bozděchová, Anna Rudolfová, Kateřina Hanáková, Miloslava Fojtová, Jiří Fajkus

**Affiliations:** 1 Mendel Centre for Plant Genomics and Proteomics, Central European Institute of Technology, Masaryk University, Kamenice 5, 62500 Brno, Czech Republic; lucie.bozdechova@ceitec.muni.cz (L.B.); hanakova.k@mail.muni.cz (K.H.); fojtova@sci.muni.cz (M.F.); 2 National Center for Biomolecular Research, Faculty of Science, Masaryk University, Kamenice 5, 62500 Brno, Czech Republic; 484185@mail.muni.cz; 3 Institute of Biophysics, Czech Academy of Sciences, Královopolská 135, 61200 Brno, Czech Republic

**Keywords:** *Arabidopsis thaliana*, ChIRP-MS, RNA, RNA-centric methods, RNA-protein interactions, telomerase, telomerase RNA

## Abstract

The current repertoire of methods available for studying RNA-protein interactions in plants is somewhat limited. Employing an RNA-centric approach, particularly with less abundant RNAs, presents various challenges. Many of the existing methods were initially designed for different model systems, with their application in plants receiving limited attention thus far. The Comprehensive Identification of RNA-Binding Proteins by Mass Spectrometry (ChIRP-MS) technique, initially developed for mammalian cells, has been adapted in this study for application in *Arabidopsis thaliana*. The procedures have been meticulously modified and optimized for telomerase RNA, a notable example of a low-abundance RNA recently identified. Following these optimization steps, ChIRP-MS can serve as an effective screening method for identifying candidate proteins interacting with any target RNA of interest.

## 1. Introduction

The identification and description of RNA-protein interactions in plants is largely dependent on the scope of the available methods. Most of these were originally developed for different model organisms and require substantial modification for use in plants. These methods can be divided into two groups—protein-centric and RNA-centric methods—based on the initial molecule of interest. Protein-centric approaches are more common because of the accessibility and feasibility of these methods [1]. On the other hand, it is sometimes necessary to opt for an approach that starts with the RNA itself, for instance, when searching for new RNA-interacting proteins or when antibodies for a protein of interest are not available. The native conditions of in vivo experiments are essential to avoid the drawbacks of in vitro systems, such as non-native RNA conformations or the absence of posttranscriptional modifications that occur in living cells.

One strategy that fits these criteria is the comprehensive identification of RNA-binding proteins by mass spectrometry (ChIRP-MS) [2] that emerged from the original chromatin isolation by RNA purification (ChIRP) sequencing-based method for the determination of genomic DNA binding sites of lncRNAs [3]. It became the first tool for binding defined parts of chromatin interacting with lncRNAs in vivo [4], and it should be applicable to both highly expressed and low abundance RNAs [2]. The (dis)advantage of ChIRP-MS is that it works with endogenously expressed RNA, reflecting the natural status of cells.

One of the low copy number lncRNAs is telomerase RNA (TR), which creates a scaffold for the formation of an RNA-protein (RNP) complex preventing chromosome ends, termed telomeres, from critical shortening leading to loss of genetic information. The telomerase catalytic core is composed of two parts: telomerase reverse transcriptase (TERT), which synthesizes telomeric repeats at the ends of eukaryotic chromosomes, and TR, which contains a template for repeat synthesis [5]. It was calculated that there are ca. 1000 molecules of hTR per cell and only approximately 200 molecules are folded into the active complex in immortalized human cell lines (HEK 293T, HeLa) [6], thus the amount of TR may be even lower in normally dividing tissue. The size of the TR subunit varies a lot across organisms, ranging from 140 nucleotides in ciliates to fungal TRs exceeding 2400 nucleotides [7]. This variation therefore suggests that different TRs have different spaces for species-specific RNA-protein interactions. In the model organism *Arabidopsis thaliana*, the telomerase RNA (AtTR) is 268 nucleotides long [8,9] and was originally described as a hypoxia-responsive lncRNA [10]. The number of confirmed interacting partners of AtTR (in addition to AtTERT) is currently limited to the plant homologue of dyskerin (AtCBF5) [8,11]. Therefore, it is likely that many other protein components of telomerase bind via the AtTR scaffold, which remains to be identified. If these are found, it will then be possible to elucidate the composition, structure, and regulatory principles governing the reversible regulation of plant telomerase [12].

**Figure 1:**
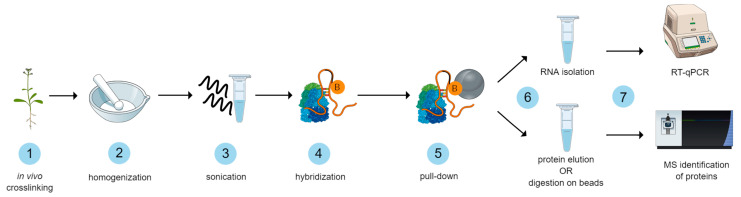
ChIRP-MS workflow in plants. Fresh plant material is crosslinked with formaldehyde, homogenized, and tissues are lysed. The fragmentation of the sample content is improved by sonication. Then, hybridization with biotin-labeled probes is performed, followed by pull-down with streptavidin paramagnetic beads. Pulled-down proteins can be either digested directly on the beads or eluted for the purpose of in-gel digestion followed by MS analysis or Western blot. The small fraction of beads after pull-down is subjected to RNA isolation and evaluated for the enrichment of target RNA by RT-qPCR. The figure was created using the Mind the Graph Platform, available at www.mindthegraph.com (accessed on 25 September 2023).

The ChIRP-MS method workflow depicted in Figure 1 starts with formaldehyde crosslinking of seedlings. The following optimization steps include homogenization, hybridization of locked nucleic acid (LNA) probes, RNA isolation, and sample preparation for MS analysis. The specificity of the method is achieved by specific antisense LNA probes with attached biotin that hybridize to target RNA and are captured with streptavidin beads together with crosslinked molecules that occur in their proximity. Moreover, probe design depends on the primary sequence and does not require knowledge of RNA structure [3]. In this study, we focused on optimizing the individual steps of this method to be applicable to plants.

## 2. Results

### 2.1. Homogenization, Isolation of Nuclei, and Sonication

**Figure 2:**
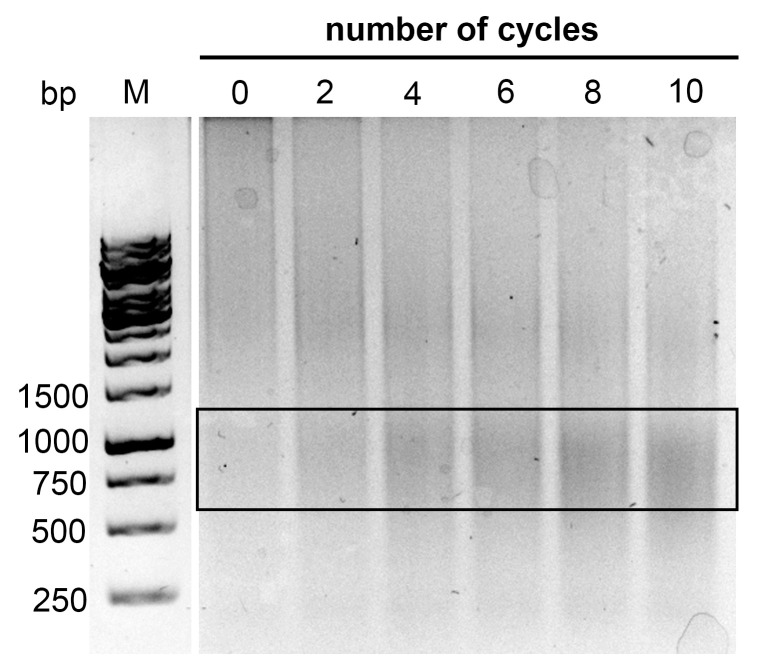
Optimization of the sonication step in nuclei lysates. The number of sonication cycles sufficient for fragmentation of the nucleic acid extract was determined by testing samples exposed to 0, 2, 4, 6, 8, and 10 cycles. A suitable fragment length between 750 bp and 1 kb appeared after 4 cycles of sonication (in the black rectangle). M—Gene Ruler 1 kb DNA ladder (Thermo Fisher Scientific, Waltham, MA, USA).

In the first step of optimization, whole cell lysate was used as a starting material for subsequent pull-down procedures. In both tested samples, RNA fragments were present in the soluble fraction (Figure S1, lanes 2 and 4). The large volume of the sample (20 mL) caused difficulties with sonication, and thus there were unequal yields of nucleic acids from independent experiments using the same amount of seedlings. To reduce the sample volume, we focused on the isolation of the nuclear fraction [13], where the target RNA is predominantly localized. The sonication step was then easier and was possible to handle in approximately 6 min. The conditions for sonication were optimized by testing different numbers of sonication pulses, from 0 to 10 cycles (Figure 2). Ten µL of sonicated lysates were directly visualized on agarose gels. The samples after four and six cycles of sonication had the lowest number of pulses to show a fragment length corresponding to the desired size, ranging from 750 bp to 1 kb (Figure 2, area indicated by a black rectangle); thus, five cycles of sonication were selected for the final protocol. As such, it can be concluded that the isolation of nuclei followed by sonication makes the procedure easier and more reproducible.

### 2.2. RNA Extraction and RT-qPCR

RNA extraction is a crucial step in sample preparation that can determine the efficiency and specificity of RNA-protein pull-down experiments using LNA probes. Using the original procedure [2], where TRI reagent extraction is combined with the use of a RNeasy Plant Mini Kit (Qiagen, Hilden, Germany), we were not able to reproducibly isolate RNA, neither from the input nor the bound fraction. Therefore, a combination of TRI reagent extraction with ethanol precipitation was used for the isolation of RNA. This approach gave us consistently better yields of total RNA from the input fraction in cases where the concentration was measurable. For the isolation of RNA enriched on beads, 10% of the sample was used. The concentration of RNA in the pull-down fraction was below the detection of nanodrops, and thus the amount of RNA added to the reverse transcription is described in terms of a relative proportion of the input fraction.

**Figure 3:**
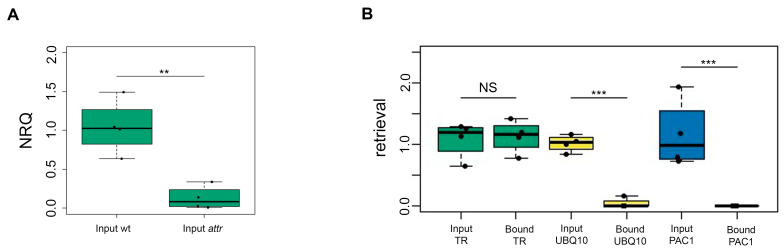
RT-qPCR confirms binding specificity for the target RNA. (**A**) Comparison of relative AtTR abundance (2^−ΔCt^) in inputs of wt and *attr* mutant plants. The level of AtTR in *attr* was significantly reduced (** = *p*-value < 0.01). NRQ, normalized relative quantities. (**B**) RNA retrieval in bound fractions, comparing the binding efficiencies of AtTR, *UBQ10,* and *PAC1* transcripts. Beads specifically bound AtTR, while the unspecific binding of the *UBQ10* and *PAC1* transcripts did not occur. The efficiency of the AtTR pull-down reached ≈100%. Significance (*** = *p*-value < 0.001) was tested by *t*-test with Welch correction. The plots were created in RStudio version 4.3.1. Experiments were performed in four biological replicates, and each sample was analyzed in technical triplicates.

The relative abundance of AtTR was tested in inputs of wild-type (wt) and *attr* samples, with the level of TR in seedlings of mutant plants significantly lower compared to wt. The AtTR signal was normalized to *UBQ10* and *PAC1* reference transcripts, and the results from four independent experiments showed that the level of TR was significantly reduced in mutant plants (Figure 3A).

For further experiments, wt seedlings were used. Specific enrichment of AtTR in the bound fraction was calculated as the ratio of relative abundances of AtTR in the bound and input fractions, with the amount of target in the input defined as 100%. The amount of AtTR in the bound fractions showed approximately 100% retrieval of AtTR, whereas transcripts of reference genes *UBQ10* and *PAC1* did not bind (Figure 3B). These results demonstrated binding specificity when using LNA probes for pull-down experiments, which is in accordance with previous studies [2,11]. Altogether, these results provide evidence that LNA pull-down is efficient and specific for targeting a low-abundance RNA of interest.

### 2.3. Protein Elution vs. On-Bead Digestion

Proteins associated with the RNA of interest can be eluted using biotin elution buffer [14], separated on SDS-PAGE, followed by in-gel digestion, or digestion can be performed directly on the beads. The first option provides an opportunity to analyze selected bands; however, less abundant proteins may be lost. We therefore opted to use SDS-PAGE analysis of eluted proteins first, which served as a control point for the presence of proteins and successful pull-down. Following this, we used direct digestion of the total protein population bound to the beads.

**Figure 4:**
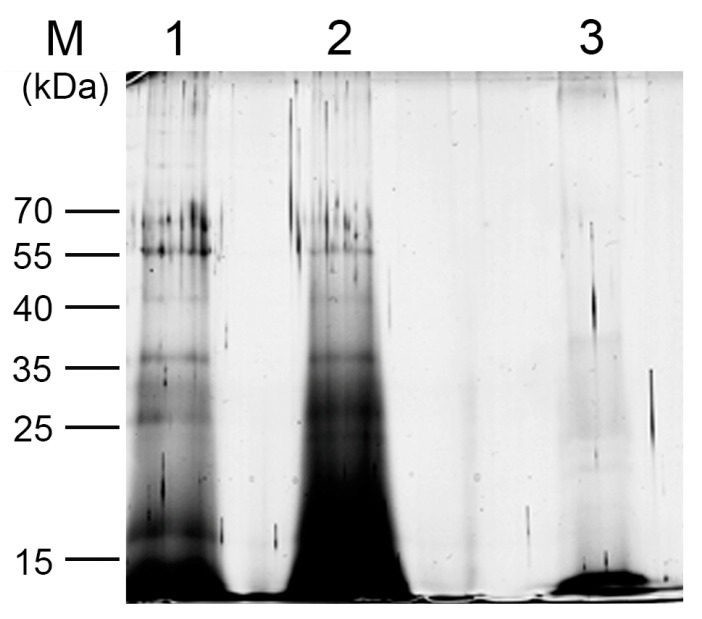
Protein elution provides a different pattern for wild-type and mutant plants on SDS-PAGE. Proteins were eluted from the beads, separated by SDS-PAGE, and silver stained. The signal from the wt sample incubated with antisense probes (lane 1) differed from that of *attr* mutant plants (lane 3). Wild-type plants incubated with sense probes (lane 2) showed a high background and thus were not applicable as a negative control. M—PageRuler™ Prestained Protein Ladder (Thermo Fisher Scientific).

To simplify the procedure, the samples on the beads were mixed directly with the loading buffer, resolved on an SDS-PAGE gel, and silver-stained. Samples were difficult to assess because of background noise from streptavidin beads (Figure S2). To compensate for this, proteins were first eluted from the beads as in [2], resolved on SDS-PAGE, and silver stained (Figure 4). To confirm that proteins are indeed TR-associated in wt plants and not simply adventitiously co-purified (Figure 4, lane 1), another negative control was included. LNA sense probes (Table S1) with the same sequence as the single-stranded regions of telomerase RNA were used for pull-down. We assumed that these probes do not hybridize with TR as they are not complementary to the target. Therefore, this negative control sample after pull-down would not contain the specific TR-interacting partners as assumed for the sample pulled-down with antisense probes. Unfortunately, sense probes still provided a strong signal after silver staining (Figure 4, lane 2), which could distort the resulting mass spectrometric analysis compared to the *attr* mutant plants (Figure 4, lane 3). Therefore, we opted to use *attr* mutant plants as a negative control, which have a markedly reduced level of TR, unlike pull-down experiments using sense probes. These results are in agreement with [2], where the authors recommend the use of mutant lines as a negative control instead of the sense probes, which feature a high background. Identification of proteins from excised gel bands using LC-MS/MS was not as clear as expected (Table 1), thus the final mass spectrometric analysis was performed using samples directly digested on the beads and analyzed by MS.

### 2.4. TR-Binding Proteins Identified by Mass-Spectrometry Analysis

In mass spectrometry analyses of proteins following direct digestion of the beads, 608 proteins were identified from 3 replicates (Table 1). Comparison of proteins represented in wt and *attr* samples revealed 162 proteins as markedly upregulated in wt (protein hits were filtered by selecting the log2 ratio of threshold 4 for upregulated proteins) or present in the wt sample and not in the *attr* negative control.

**Figure 5:**
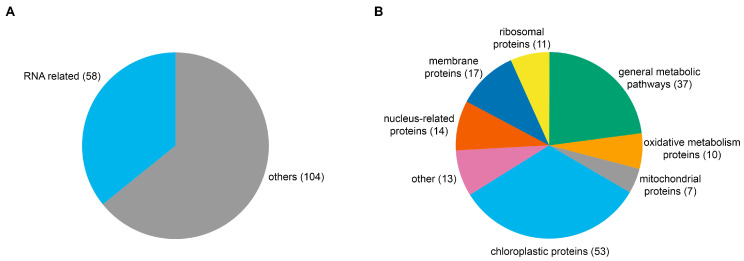
Overview of TR-associated proteins identified by LC-MS/MS. (**A**) Based on gene ontology, about one-third of the identified proteins were related to RNA. (**B**) Proteins detected by LC-MS/MS were divided based on their occurrence and function into several groups connecting to different cellular pathways.

About one-third of the upregulated proteins were identified as RNA-related, based on their gene ontology and molecular function (Figure 5A).

The obtained set of proteins was divided into several groups based on available information about their occurrence and/or function (Figure 5B). The majority of identified proteins were related to chloroplasts, mitochondria, oxidation (mostly peroxidases), general metabolism (mostly carbohydrate metabolism), and various membranes or ribosomes. Nucleus-related proteins were also identified, which are of primary interest for future evaluations, considering that telomerase RNA is predominantly localized in the nucleus.

## 3. Discussion

RNA-centric approaches have rarely been used for the detection of candidate proteins in plants. In this work, the ChIRP-MS method was chosen as a tool for identifying potential interacting partners of telomerase RNA. The method was originally designed for mammalian cells; thus, the main goal was to adjust the procedure for use in the model plant *A. thaliana*, as the repertoire of such methods adapted to plant specificities is limited. An RNA-centric approach may represent an elegant escape from a dead end, particularly when suitable antibodies are lacking for the use of protein-centric methods. The plant-specific ChIRP-MS protocol prepares the ground for future interactor screens, making the method more accessible for plant scientists.

The lysis procedure is simple when using elementary lysis buffer [2] for mammalian cells that lack a cell wall. However, considering the differences of plant cells, optimization of homogenization and the determination of the proper volume of lysis buffer were necessary steps. The large volume of buffer employed requires great consumption of inhibitors and LNA probes, and the target RNA is diluted to a very low concentration, even below the detection limit of RT-qPCR amplification, which caused inconveniences for later RNA extraction and confirmation of enrichment. Telomerase RNA is predominantly present in the nucleus; therefore, we took advantage of the standard plant nuclei isolation protocol to remove other cellular organelles and obtain less diluted RNA.

The important part of the procedure is the selection of negative control, which enables the removal of unspecific protein hits. In the previous publication [2], authors used RNase A-treated samples as a negative control, bringing with it a potential risk of contaminating other samples and degrading the target RNA. Two negative controls were tested in our experimental setup—sense LNA probes and *attr* mutant plants with knockdown telomerase RNA. Because of the high background in samples pulled down by the sense probes, *attr* mutant plants were used as a preferred negative control, similarly as in [11].

Specific enrichment of the target RNA was achieved compared to negative controls, despite its low concentration. We were able to obtain approximately 100% of the presented TR, while the unspecific capture of *UBQ10* and *PAC1* transcripts did not occur.

Probably the most limiting factor in the robustness of the method was the low number of telomerase RNA transcripts, which required a huge amount of entry material and likely led to a variable output from the MS analysis. Because of the low yield of protein hits obtained from in-gel digestion analysis, on-bead digestion was performed in three replicates and led to the detection of several hundred proteins. Thus SDS-PAGE analysis served more as a confirmation of the applicability of the optimized protocol and successful protein pull-down. Likely due to lower stringency in the case of a low-abundant RNA, there were common proteins related to general metabolism and most prominently to chloroplasts, as their complete removal during nuclei purification could not be reached. Further sorting of hits was thus inevitable; however, it still provided a shortlist of biologically relevant candidates.

The *attr* control was also not completely TR-free as opposed to the RNAse-treated control [2], which may be another reason for the relatively small fold changes in the identified hits. Nevertheless, the aim of the method was never to directly prove individual interactions but rather to obtain a group of candidate RNA-associated proteins.

Altogether, the ChIRP-MS method was successfully modified for the plant model *Arabidopsis thaliana,* and this was used for the identification of potential interacting partners of AtTR. The identification rate of the candidate proteins depends on the amount of target RNA in the cells and the approach for sample preparation for LC-MS/MS analysis. Based on this work, we believe that ChIRP-LC-MS/MS is a useful tool as a screening method for the identification of candidate RNA-interacting proteins not only in human cells but also in plants. This method and other RNA-centric methods will be of increasing importance due to rapidly expanding research on the roles of non-coding RNAs. Optimization of ChIRP-MS using a particularly challenging target RNA—the low abundance AtTR—promises to be applicable to the current demands of research on other functional plant RNAs, in particular ncRNAs.

## 4. Materials and Methods

### 4.1. Plant Cultivation

*Arabidopsis thaliana* ecotype Col-0 (wt) and *attr* mutant (SALK_076745) seeds were sterilized with 1 mL of 70% ethanol for 1 min. Seeds were dried on filter paper and sown on a 10 cm square Petri dish on ½ MS agar medium (0.22% Murashige and Skoog Basal Salt Mixture (Duchefa, Haarlem, The Netherlands); 1% sucrose; 0.8% plant agar (Duchefa); 1 M NaOH for pH = 5.8 adjustment). The seeds were subjected to stratification in the dark at 4 °C for 48 h and then positioned vertically in controlled long-day conditions: 16/8 h day/night photoperiod, 21 °C, light intensity 150 µmol·m^−2^·s^−1^. 7-day-old seedlings were used for all experiments.

### 4.2. Crosslink

2 g of 7-day-old seedlings were submerged in 50 mL of 1% formaldehyde and put into a vacuum desiccator for 10 min with occasional mixing. The reaction was stopped by adding 2 M glycine (final concentration: 0.125 M) and applying vacuum for another 10 min. The seedlings were captured using two layers of silk and washed three times with pre-cooled Milli-Q water. Residual water was removed by blotting with filter paper. The seedlings were then wrapped in aluminum foil and immediately placed into liquid nitrogen. Following this, they were stored at −80 °C for further use.

### 4.3. Whole Cell Lysate Preparation

2 g of 7-day-old seedlings were homogenized, maintaining a ratio of 1 mL of buffer per 100 mg of material [2,11] and then sonicated with a Misonix S-4000 sonicator with amplitude 15; 5 s ON; 3 s OFF for 6 min, with pauses after each 1 min. 10 µL of lysate was separated into soluble and insoluble fractions and used for DNA isolation as in [2] to check if DNA is present in the soluble fraction. DNA isolation was performed with a Purelink™ Quick PCR Purification Kit (Invitrogen, Waltham, MA, USA) according to the manufacturer’s instructions. Isolated DNA fragments were then resolved on a 1% agarose gel.

### 4.4. Homogenization of Plant Material and Isolation of Nuclei

The isolation of nuclei was carried out based on [13]. All buffers were prepared using RNase-free, DMPC-treated water. Leupeptin (Serva, Heidelberg, Germany), Pepstatin A (Roth, Karlsruhe, Germanys), and PMSF (Serva) were added to prevent protein degradation, while RNaseOUT™ Recombinant Ribonuclease Inhibitor (Invitrogen) was used to protect RNA.

Ten g of crosslinked plant material was gently homogenized in a mortar with liquid nitrogen. The resulting powder was divided into two precooled 50 mL falcon tubes filled with Extraction Buffer 1 (0.4 M sucrose, 10 mM Tris-HCl pH 8.0, 5 mM β-mercaptoethanol, 0.1 µg⋅µL^−1^ Leupeptin, 0.1 µg⋅µL^−1^ Pepstatin A, 0.1 mM PMSF) and vortexed to solubilize the powder. The homogenate was then filtered through two layers of nylon mesh (20 × 20 cm; pore size 162 µm) into a beaker placed on ice via a pre-chilled funnel. The liquid was split equally into two 50 mL falcon tubes placed on ice, and these were centrifuged at 2880× *g* for 20 min at 4 °C. The supernatant was removed, and the pellets were homogenized in 5 mL of Extraction Buffer 2 (0.25 M sucrose, 10 mM Tris-HCl pH 8.0, 10 mM MgCl_2_, 0.1% Triton X-100 (Sigma), 5 mM β-mercaptoethanol, 0.1 µg⋅µL^−1^ Leupeptin, 0.1 µg⋅µL^−1^ Pepstatin A, 0.1 mM PMSF) by pipetting up and down and combined in one 15 mL falcon tube. The samples were then centrifuged at 12,000× *g* for 10 min at 4 °C. This step was repeated 5–6 times using 5 mL of Extraction Buffer 2 until the pellets became white and the supernatant stopped taking in chlorophyll. The pellet was then resuspended in 2 mL of Extraction Buffer 2 and carefully layered on 2 mL of Extraction Buffer 3 (1.7 M sucrose, 10 mM Tris-HCl pH 8.0, 2 mM MgCl_2_, 0.15% Triton X-100, 5 mM β-mercaptoethanol, 0.1 µg⋅µL^−1^ Leupeptin, 0.1 µg⋅µL^−1^ Pepstatin A, 0.1 mM PMSF). The samples were centrifuged at 16,000× *g* for 1 h at 4 °C, and the supernatant was removed. Finally, isolated nuclei were resuspended in 2.5 mL of Nuclei Lysis Buffer (0.1% SDS, 50 mM Tris-HCl pH 8.0, 10 mM EDTA, 0.1 µg⋅µL^−1^ Leupeptin, 0.1 µg⋅µL^−1^ Pepstatin A, 0.1 mM PMSF, 6 mM DTT, 1 µL⋅ml^−1^ RNAseOUT, 20 µL⋅ml^−1^ Plant protease inhibitor mixture (Sigma-Aldrich, St. Louis, MO, USA)).

### 4.5. Sonication

Samples were split equally into three 1.5 mL tubes and sonicated in a Diagenode UCD-200 instrument over 5 cycles (medium power mode, 15 s ON, 1 min OFF) to improve the lysis of nuclei. Samples were combined back together into one tube, and then 10 µL of the sample was resolved on a 1% agarose gel to verify the fragmentation. The optimal length of fragments was 1–2 kb or less, as published in [2].

### 4.6. Hybridization and Pull-Down

Sonicated chromatin was centrifuged at 13,000× *g* for 10 min at 4 °C, and then the supernatant was transferred into a new tube. Ten µL of the sample were removed as an input fraction for the isolation of total RNA. Thirty µL of Dynabeads™ MyOne™ Streptavidin C1 beads (Thermo Fisher Scientific) were pre-washed three times with 100 µL Nuclei Lysis Buffer on a magnetic stand and transferred into the lysates. The mixed samples were precleared by rotating in a hybridization oven for 30 min at 42 °C. After preclearing, the supernatant was transferred to a new tube, and 2 volumes of freshly prepared and prewarmed hybridization buffer (750 mM NaCl, 1% SDS, 50 mM Tris-HCl pH 7.0, 1 mM EDTA, 15% formamide, 1 mM PMSF, 6 mM DTT, and 1 µL⋅ml^−1^ RNAseOUT) were added. One µL of LNA antisense probes (100 µM; mix of 4 probes; Table S1) was added to the samples, and they were rotated overnight at 42 °C in a hybridization oven. 100 µL of paramagnetic C1 beads were washed three times with 100 µL Nuclei Lysis Buffer, mixed with the precleared samples, and incubated for 45 min at 42 °C on the rotator. The supernatant was discarded, and the beads were washed three times with 500 µL (five times the initial volume of the beads) of Wash Buffer (2× SSC, 0.5% SDS, 1 mM PMSF, 5 mM DTT) and three times with Wash Buffer without SDS (to avoid contamination of MS samples), rotating the samples for 5 min in each round. 10 µL of the last wash suspension (bound fraction) was used for RNA isolation. The supernatant was removed, and the beads were resuspended in 30 µL of SDS-free Wash Buffer and stored at −80 °C before MS analysis.

### 4.7. RNA Extraction

Input and bound fractions were topped up to 100 µL with pK Buffer (100 mM NaCl, 10 mM Tris-HCl pH 7.0, 1 mM EDTA, 0.5% SDS, and 5% *v*/*v* of 20 mg⋅ml^−1^ proteinase K). Samples were incubated at 50 °C for 45 min with 300 rpm mixing. The beads were then heated at 95 °C for 10 min to complete de-crosslinking. The samples were then mixed with 1 mL of TRI reagent^®^ (Molecular Research Center, Inc., Cincinnati, OH, USA) and incubated for 5 min at room temperature. 100 µL of BCP (1-bromo-3-chloropropane) (Molecular Research Center, Inc.) were added, and samples were mixed for 15 s, incubated at room temperature for 15 min, and then centrifuged at 12,000× *g* for 15 min at 4 °C. The upper aqueous phase containing RNA was transferred to a fresh tube. RNA was precipitated by adding 2.5 volumes of cooled 96% ethanol, 0.1 volume of cooled 3 M sodium acetate, and 1 µL of RNA-grade glycogen (Thermo Fisher Scientific) at –20 °C overnight. The samples were centrifuged (13,000× *g* for 1 h at 4 °C), washed with 250 µL of cooled 75% ethanol, and centrifuged again (13,000× *g* for 30 min at 4 °C). Ethanol was carefully removed, and the samples were left to dry out for 5–10 min. The extracted RNA was then diluted in 10 µL of RNAse-free water.

### 4.8. RT-qPCR

To check the level of transcripts in wt and *attr* plants, along with verifying the enrichment of telomerase RNA, RT-qPCR was used. One µL of isolated RNA was used for the reverse transcription using SuperScript™ II Reverse Transcriptase (Invitrogen) and random nonamers (2.5 µM; Sigma-Aldrich) as primers according to the manufacturer’s instructions. cDNA was diluted twofold before use.

qPCR was performed using a Rotor-Gene 6000 instrument (Qiagen) as follows: hold 94 °C/15 min; 35 cycles 95 °C/20 s, 57 °C/30 s, 72 °C/30 s; hold 72 °C/3 min; melt 50 to 99 °C by increasing by 1 °C every 5 s. Each reaction (20 µL) comprised 10 µL of 2× concentrated FastStartTM SYBR^®^ Green Master (Roche, Basel, Switzerland), 8 µL of DMPC water, 0.5 µL of each primer (10 µM; for sequences see Table S1), and 1 µL of diluted cDNA.

The C_t_ values were determined using Rotor-Gene Q Series Software version 2.2.2. The enrichment of TR transcripts in wt compared to the *attr* mutant was calculated from four independent experiments with two reference genes (*UBQ10* and *PAC1*) and normalized to relative quantities of TR,

NRQ = 2^−(C_t_(AtTR)−C_tm_(AtTR))^/(2^−(C_t_(*PAC1*)−C_tm_(*PAC1*))^ × 2^−(C_t_(*UBQ10*)−C_tm_(*UBQ10*))^)^0.5^,

with C_tm_ being the mean of four replicates in wt input, as in [15]. The amount of target TR was also calculated from four independent experiments and represented as RNA retrieval (ratios of 2^C_t_^ abundances of bound and input fractions). The significance was *t*-tested or *t*-tested with Welch correction if the variances of the compared groups differed based on the F-test. A graphical output was created using RStudio [16], R version 4.2.2, and the following packages: openxlsx [17], tidyverse [18], dplyr [19], ggsignif [20], and ggpubr [21].

### 4.9. Protein Elution and SDS-PAGE

Based on the original procedure published in [2], proteins were eluted from the beads and analyzed by polyacrylamide gel electrophoresis, followed by mass spectrometry. The beads were resuspended in 100 µL of biotin elution buffer (12.5 mM D-biotin, 7.5 mM HEPES, pH = 7.5; 75 mM NaCl; 1.5 mM EDTA; 0.15% SDS; 0.075% sarcosyl; 0.02% Na-deoxycholate), mixed for 20 min at room temperature, and subsequently for 10 min at 65 °C. The supernatant was transferred into a new tube, and the beads were eluted again with 100 µL of biotin elution buffer. Both eluents were combined, and proteins were precipitated by adding 50 µL of trichloroacetic acid (25% *v*/*v*) at 4 °C overnight. Then, the samples were centrifuged at 16,000× *g* for 30 min at 4 °C, and the protein pellet was washed twice with cold acetone. The pellets were air-dried for 1 min and resuspended in 40 µL of 1× Laemmli buffer. Samples were resolved on a 12% polyacrylamide gel at 135 V for 1.5 h.

### 4.10. Gel Silver Staining

SDS-PAGE gel was fixed in 50% ethanol with a 10% acetic acid solution overnight and stained with the ProteoSilver Plus Silver Stain Kit (Sigma-Aldrich) according to the manufacturer’s instructions.

### 4.11. In-Gel Digestion

Selected 1D gel bands were excised manually after destaining and washing. Each band was subjected to protein reduction (10 mM DTT in 25 mM NH_4_HCO_3_, 45 min, 56 °C, 750 rpm) and alkylation (55 mM IAA in 25 mM NH_4_HCO_3_, 30 min, room temperature, 750 rpm). After further washing with 50% ACN/NH_4_HCO_3_ and pure ACN, gel pieces were incubated with 125 ng trypsin (sequencing grade; Promega, Madison, WI, USA) in 50 mM NH_4_HCO_3_. Trypsin digestion was performed for 2 h at 37 °C on a Thermomixer (750 rpm; Eppendorf, Hamburg, Germany). Tryptic peptides were then extracted into LC-MS vials using 2.5% formic acid (FA) in 50% ACN with the addition of polyethylene glycol (final concentration 0.001%) [22] and concentrated in a SpeedVac concentrator (Thermo Fisher Scientific).

### 4.12. Direct Protein Digestion on the Beads

Beads with bound proteins were mixed with 2% SDS and heated to 50 °C for 20 min. Proteins were reduced using 500 mM DTT at 95 ℃ for 15 min. The resulting protein solutions were subjected to filter-aided sample preparation (FASP) as described elsewhere [23] using 0.75 μg of trypsin (sequencing grade; Promega). Peptides were then cleaned by liquid-liquid extraction (3 iterations) using water-saturated ethyl acetate [24]. Cleaned FASP eluates were evaporated completely in a SpeedVac concentrator (Thermo Fisher Scientific). The resulting peptides were analyzed by LC-MS/MS.

### 4.13. LC-MS/MS Analysis of Peptides

LC-MS/MS analyses of all peptide mixtures were performed using a RSLCnano system connected to an Orbitrap Fusion Lumos mass spectrometer (Thermo Fisher Scientific). Prior to LC separation, tryptic digests were concentrated and desalted in-line using a trapping column (Acclaim™ PepMap™ 100 C18, dimensions 300 μm ID, 5 mm long, 5 μm particles, Thermo Fisher Scientific). After washing the trapping column with 0.1% FA, peptides were eluted (flow rate: 300 nL/min) onto an analytical column (Aurora C18, 75 μm ID, 250 mm long, 1.6 μm particles, Ion Opticks, Melbourne, Australia) using a 70 min gradient program (mobile phase A: 0.1% FA in water; mobile phase B: 0.1% FA in 80% ACN). Equilibration of the trapping column and the analytical column was performed prior to sample injection via a sample loop. The analytical column outlet was directly connected to the Digital PicoView 550 (New Objective, Littleton, MA, USA) ion source with a sheath gas option. An ABIRD (Active Background Ion Reduction Device, ESI Source Solutions, Woburn, MA, USA) was also installed.

Data were acquired in data-independent acquisition mode (DIA-MS). The survey scan covered *m*/*z* 350–1400 at a resolution of 60,000 (at *m*/*z* 200) with an AGC target value of 1.2 × 10^6^ and a maximum injection time of 55 ms. HCD MS/MS (27% relative fragmentation energy) were acquired in the range of *m*/*z* 200–2000 at 30,000 resolution with a target value of 5 × 10^5^. The maximum injection time for MS/MS was 55 ms. Overlapping window patterns in *m*/*z* ranging from 400 to 850 were used as isolation window placements.

DIA data were processed in DIA-NN (version 1.8) [25] using a modified cRAP database (based on http://www.thegpm.org/crap/; accessed on 19 October 2023, 112 sequences in total) and the UniProtKB protein database for *Arabidopsis thaliana* (downloaded 16 October 2022, number of protein sequences: 27,487). No optional, carbamidomethylation as a fixed modification, and trypsin/P enzyme with 1 allowed missed cleavage and peptide length 7–30 were set during the library preparation. The false discovery rate (FDR) control was set to 1% FDR. MS1 and MS2 accuracies as well as scan window parameters were set based on the initial test searches (median value from all samples ascertained parameter values). MBR was switched on.

Reported protein intensities were further processed using the software container environment (https://github.com/OmicsWorkflows; accessed on 19 October 2023). The processing workflow is available upon request. Briefly, it covered: (a) removal of decoy hits and contaminant protein groups; (b) protein group intensities in log2 transformation; and (c) loessF normalization.

## Figures and Tables

**Table 1:** Number of proteins identified by LC-MS.

Digestion Type	Replicate Number	Detected Proteins	Unique Proteins
In-gel	1	121	n.d.
On-beads	1	527	
	2	424	608 *
	3	357	

* Unique proteins from all three replicates.

## Data Availability

The mass spectrometry proteomics data have been deposited to the ProteomeXchange Consortium via the PRIDE [26] partner repository with the dataset identifier “PXD046839”.

## Supplementary Materials

The following supporting information can be downloaded at: https://www.mdpi.com/article/10.3390/plants13060850/s1, Figure S1: Whole cell lysates divided into insoluble and soluble fractions (containing chromatin) and resolved on agarose gel; Figure S2: Proteins eluted by direct boiling of the beads, resolved on SDS-PAGE gel, and silver stained; Table S1: List of oligonucleotides used for pull-down assays and qPCR.

## References

[1] Li Y., Liu S., Cao L., Luo Y., Du H., Li S., Zhang Z., Guo X., Tian W., Wong C.C. (2021). CBRPP: A New RNA-Centric Method to Study RNA-Protein Interactions. RNA Biol..

[2] Chu C., Chang H.Y. (2018). ChIRP-MS: RNA-Directed Proteomic Discovery. Methods Mol. Biol..

[3] Chu C., Quinn J., Chang H.Y. (2012). Chromatin Isolation by RNA Purification (ChIRP). J. Vis. Exp. JoVE.

[4] Chu C., Chang H.Y. (2016). Understanding RNA-Chromatin Interactions Using Chromatin Isolation by RNA Purification (ChIRP). Methods Mol. Biol..

[5] Greider C.W., Blackburn E.H. (1989). A Telomeric Sequence in the RNA of Tetrahymena Telomerase Required for Telomere Repeat Synthesis. Nature.

[6] Xi L., Cech T.R. (2014). Inventory of Telomerase Components in Human Cells Reveals Multiple Subpopulations of hTR and hTERT. Nucleic Acids Res..

[7] Podlevsky J.D., Chen J.J.-L. (2016). Evolutionary Perspectives of Telomerase RNA Structure and Function. RNA Biol..

[8] Fajkus P., Peška V., Závodník M., Fojtová M., Fulnečková J., Dobias Š., Kilar A., Dvořáčková M., Zachová D., Nečasová I. (2019). Telomerase RNAs in Land Plants. Nucleic Acids Res..

[9] Song J., Logeswaran D., Castillo-González C., Li Y., Bose S., Aklilu B.B., Ma Z., Polkhovskiy A., Chen J.J.-L., Shippen D.E. (2019). The Conserved Structure of Plant Telomerase RNA Provides the Missing Link for an Evolutionary Pathway from Ciliates to Humans. Proc. Natl. Acad. Sci. USA.

[10] Wu J., Okada T., Fukushima T., Tsudzuki T., Sugiura M., Yukawa Y. (2012). A Novel Hypoxic Stress-Responsive Long Non-Coding RNA Transcribed by RNA Polymerase III in Arabidopsis. RNA Biol..

[11] Song J., Castillo-González C., Ma Z., Shippen D.E. (2021). Arabidopsis Retains Vertebrate-Type Telomerase Accessory Proteins via a Plant-Specific Assembly. Nucleic Acids Res..

[12] Fajkus J., Fulnecková J., Hulánová M., Berková K., Ríha K., Matyásek R. (1998). Plant Cells Express Telomerase Activity upon Transfer to Callus Culture, without Extensively Changing Telomere Lengths. Mol. Gen. Genet. MGG.

[13] Adamusová K., Khosravi S., Fujimoto S., Houben A., Matsunaga S., Fajkus J., Fojtová M. (2020). Two Combinatorial Patterns of Telomere Histone Marks in Plants with Canonical and Non-Canonical Telomere Repeats. Plant J..

[14] Chu C., Zhang Q.C., da Rocha S.T., Flynn R.A., Bharadwaj M., Calabrese J.M., Magnuson T., Heard E., Chang H.Y. (2015). Systematic Discovery of Xist RNA Binding Proteins. Cell.

[15] González-Bermúdez L., Anglada T., Genescà A., Martín M., Terradas M. (2019). Identification of Reference Genes for RT-qPCR Data Normalisation in Aging Studies. Sci. Rep..

[16] R Core Team (2022). R: A Language and Environment for Statistical Computing.

[17] Schauberger P., Walker A., Braglia L., Sturm J., Garbuszus J.M., Barbone J.M. (2023). Read, Write and Edit Xlsx Files. https://cran.r-project.org/web/packages/openxlsx/openxlsx.pdf.

[18] Wickham H. (2023). Package ‘Tidyverse’. https://cran.r-project.org/web/packages/tidyverse/tidyverse.pdf.

[19] Wickham H., François R., Henry L., Müller K., Vaughan D. (2023). Package ‘Dplyr’. https://dplyr.tidyverse.org/.

[20] Ahlmann-Eltze C., Patil I. (2022). Package ‘Ggsignif’. https://cran.r-project.org/web/packages/ggsignif/ggsignif.pdf.

[21] Kassambara A. (2023). Package ‘Ggpubr’. https://cran.r-project.org/web/packages/ggpubr/ggpubr.pdf.

[22] Stejskal K., Potěšil D., Zdráhal Z. (2013). Suppression of Peptide Sample Losses in Autosampler Vials. J. Proteome Res..

[23] Wiśniewski J.R., Ostasiewicz P., Mann M. (2011). High Recovery FASP Applied to the Proteomic Analysis of Microdissected Formalin Fixed Paraffin Embedded Cancer Tissues Retrieves Known Colon Cancer Markers. J. Proteome Res..

[24] Yeung Y.-G., Nieves E., Angeletti R., Stanley E.R. (2008). Removal of Detergents from Protein Digests for Mass Spectrometry Analysis. Anal. Biochem..

[25] Demichev V., Messner C.B., Vernardis S.I., Lilley K.S., Ralser M. (2020). DIA-NN: Neural Networks and Interference Correction Enable Deep Proteome Coverage in High Throughput. Nat. Methods.

[26] Perez-Riverol Y., Bai J., Bandla C., García-Seisdedos D., Hewapathirana S., Kamatchinathan S., Kundu D.J., Prakash A., Frericks-Zipper A., Eisenacher M. (2021). The PRIDE Database Resources in 2022: A Hub for Mass Spectrometry-Based Proteomics Evidences. Nucleic Acids Res..

