# Supplementary material for: Optimizing ChIRP-MS for Comprehensive Profiling of RNA-Protein Interactions in Arabidopsis thaliana: A Telomerase RNA Case Study
Source: Plants (Basel). 2024 Mar 15;13(6):850. doi: 10.3390/plants13060850 (PMC10975786; doi:10.3390/plants13060850)
Supplement: Supplementary file 1 [file plants-13-00850-s001.zip › plants-2854973-supplementary.pdf]

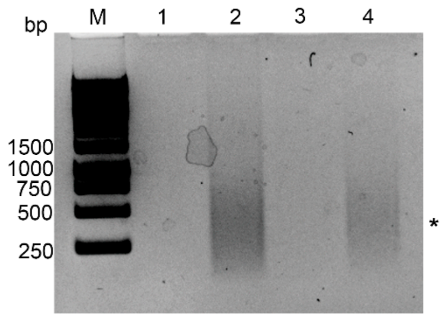

Figure S1 Comparison of soluble and insoluble fractions of whole cell lysates

Chromatin fragmentation in 20 ml of homogenate is inconsistent. Chromatin is present in soluble fractions (lane 2 and 4) of the samples but with different yields (marked with an asterisk). Lane 1 and 2 represent insoluble fractions.

M - Gene Ruler 1 kb DNA ladder (Thermo Fisher Scientific).

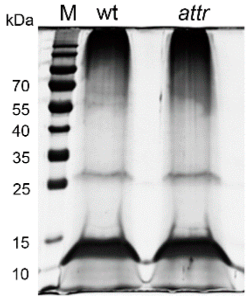

Figure S2 Protein elution by direct boiling of the beads

Protein samples were boiled directly on the beads, resolved by SDS-PAGE and silver stained. In both samples, the signal was overlaid by noise, likely originating from streptavidin beads after boiling.

M - PageRuler™ Prestained Protein Ladder (Thermo Fisher Scientific)

Table S1 List of oligonucleotides used in this study

Primer name	*sequence*	*analysis*
AtTR Fw	TTACTGGGGGTCTTAGGCCG	qPCR
AtTR Rev	GGAGGGTAAGGCGAGGAAAC	qPCR
PAC1 Fw	TCTCTTTGCAGGATGGGACAAGC	qPCR
PAC1 Rev	AGACTGAGCCGCCTGATTGTTTG	qPCR
UBQ Fw	AACGGGAAAGACGATTAC	qPCR
UBQ Rev	ACAAGATGAAGGGTGGAC	qPCR
VRP 516	AA+AT+AT+T+TG+G+G+GG/3BioTEG/	antisense; pull-down
VRP 517	A+C+C+TA+CG+A+AC+C/3BioTEG/	antisense; pull-down
VRP 518	CC+TG+G+TC+TA+CT+TT+TT/3BioTEG/	antisense; pull-down
VRP 519	TT+TT+CA+TT+ATT+TTT+CGGGGG/3BioTEG/	antisense; pull-down
Probe1	C+C+C+C+CAA+ATAT+TT/3BioTEG/	sense; pull-down
Probe2	G+GT+T+CG+T+AG+GT/3BioTEG/	sense; pull-down
Probe3	AA+AA+A+GTA+G+AC+CA+GG/3BioTEG/	sense; pull-down
Probe4	CCCCC+G+A+A+AAAT+A+AT+G+A+A+AA/3BioTEG/	sense; pull-down

+ = Affinity Plus base

/3BioTEG/ = 3′-biotin-triethyleneglycole spacer
